# Supplementary material for: Phage Engineering for Targeted Multidrug-Resistant Escherichia coli
Source: Int J Mol Sci. 2023 Jan 27;24(3):2459. doi: 10.3390/ijms24032459 (PMC10004113; doi:10.3390/ijms24032459)

Figure S1 Whole-genome comparison analysis. (A) Genome comparison between PHB20 and other phages with higher homology of nucleotide sequence. Each arrow indicated an annotated ORF. The color intensity is proportional to the sequence homology. **(B)** Primers were designed for phage tail fibers.

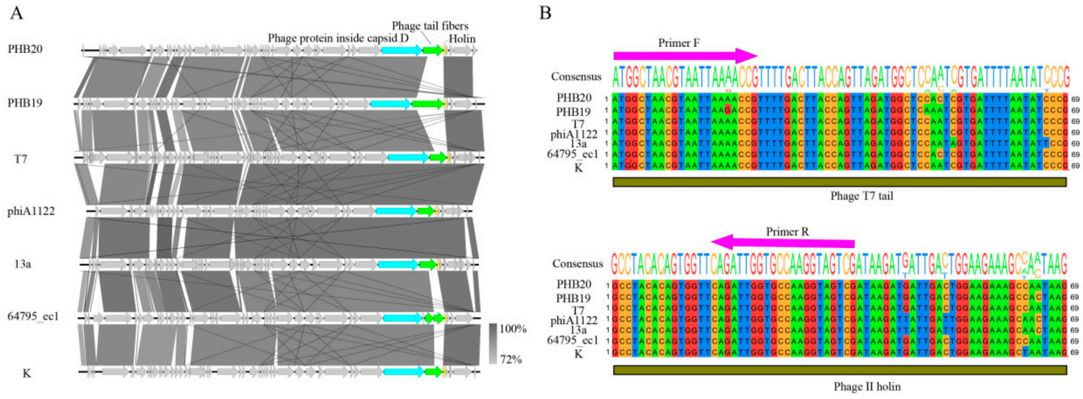

Supplement: Supplementary file 1 [file ijms-24-02459-s001.zip › Figure S1.pdf]
